# Identification of subgroups of inflammatory and degenerative MRI findings in the spine and sacroiliac joints: a latent class analysis of 1037 patients with persistent low back pain

**DOI:** 10.1186/s13075-016-1131-x

**Published:** 2016-10-13

**Authors:** Bodil Arnbak, Rikke Krüger Jensen, Claus Manniche, Oliver Hendricks, Peter Kent, Anne Grethe Jurik, Tue Secher Jensen

**Affiliations:** 1 Research Department, Spine Centre of Southern Denmark, Hospital Lillebaelt, Oestre Hougvej 55, Middelfart, 5500 Denmark; 2 Institute of Regional Health Research, University of Southern Denmark, Winsloewparken 19-3, Odense C, 5000 Denmark; 3 King Christian 10th Hospital for Rheumatic Diseases, Toldbodgade 3, Graasten, 6300 Denmark; 4 School of Physiotherapy and Exercise Science, Curtin University, Kent Street, Bentley, Perth, Western Australia 6102 Australia; 5 Department of Sports Science and Clinical Biomechanics, University of Southern Denmark, Campusvej 55, Odense M, 5230 Denmark; 6 Department of Radiology, Aarhus University Hospital, Noerrebrogade 44, Aarhus C, 8000 Denmark; 7 Nordic Institute of Chiropractic and Clinical Biomechanics, Campusvej 55, Odense M, 5230 Denmark

**Keywords:** Cluster analysis, Low back pain, Magnetic resonance imaging, Sacroiliac joints, Spondyloarthritis, Spine

## Abstract

**Background:**

The aim of this study was to investigate subgroups of magnetic resonance imaging (MRI) findings for the spine and sacroiliac joints (SIJs) using latent class analysis (LCA), and to investigate whether these subgroups differ in their demographic and clinical characteristics.

**Methods:**

The sample included 1037 patients aged 18–40 years with persistent low back pain (LBP). LCA was applied to MRI findings of the spine and SIJs. The resulting subgroups were tested for differences in self-reported demographic and clinical characteristics.

**Results:**

A five-class model was identified: Subgroup 1, ‘No or few findings’ (*n* = 116); Subgroup 2, ‘Mild spinal degeneration’ (*n* = 540); Subgroup 3, ‘Moderate to severe spinal degeneration’ (*n* = 229); Subgroup 4, ‘Moderate to severe spinal degeneration with mild SIJ findings’ (*n* = 68); and Subgroup 5, ‘Mild spinal degeneration with moderate to severe SIJ findings’ (*n* = 84). The two SIJ subgroups (Subgroups 4 and 5) had a higher median activity limitation score (Roland Morris Disability Questionnaire calculated as a proportional score: 65 (IQR 48–78)/65 (48–78)) compared with Subgroups 1–3 (48 (35–74)/57 (39–74)/57 (39–74)), a higher prevalence of women (68 % (95 % CI 56–79)/68 % (58–78)) compared with Subgroups 2 and 3 (51 % (47–55)/40 % (33–46)), a higher prevalence of being overweight (67 % (95 % CI 55–79)/53 % (41–65)) compared with Subgroup 1 (36 % (26–46)) and a higher prevalence of previous LBP episodes (yes/no: 81 % (95 % CI 71–91)/79 % (70–89)) compared with Subgroup 1 (58 % (48–67)). Subgroup 5 was younger than Subgroup 4 (median age 29 years (IQR 25–33) versus 34 years (30–37)) and had a higher prevalence of HLA-B27 (40 % (95 % CI 29–50)) compared with the other subgroups (Subgroups 1–4: 12 % (6–18)/7 % (5–10)/6 % (3–9)/12 % (4–20)). Across the subgroups with predominantly spinal findings (Subgroups 1–3), median age, prevalence of men, being overweight and previous LBP episodes were statistically significantly lower in Subgroup 1, higher in Subgroup 2 and highest in Subgroup 3.

**Conclusions:**

Five distinct subgroups of MRI findings in the spine and SIJs were identified. The results indicate that SIJ MRI findings not only can be seen as a part of the spondyloarthritis disease entity, but also are associated with age, gender and being overweight. Furthermore, the results indicate that LBP patients with SIJ MRI findings are more disabled compared with patients without SIJ MRI findings, and that moderate to severe spinal degeneration and/or SIJ MRI findings may be associated with recurrent pain.

**Electronic supplementary material:**

The online version of this article (doi:10.1186/s13075-016-1131-x) contains supplementary material, which is available to authorized users.

## Background

Low back pain (LBP) is a serious and disabling health condition that is estimated to be the number-one cause of years lived with disability [1]. More knowledge about the various causes of LBP is needed to improve diagnosis and treatment. The use of magnetic resonance imaging (MRI) has increased dramatically in recent decades in an attempt to optimise the diagnostic process for persistent LBP and spondyloarthritis (SpA). Nevertheless, many uncertainties remain about the association between MRI findings and the clinical presentation of back pain [2–5].

Several MRI findings, including degenerative findings such as disc degeneration, disc herniations and vertebral endplate signal changes (i.e. Modic changes), have been associated with clinical presence of LBP. Also, and findings at the sacroiliac joints (SIJs) (i.e. sacroiliitis) have been associated with the clinical diagnosis of SpA. However, the strength of these associations is often reported to be relatively weak [2–5]. This might be because previous studies have focused on individual MRI findings [2–5], even though multiple MRI findings with varying severity are often present at the same time. In a recent cross-sectional population study of over 1000 people, some degree of lumbar disc degeneration was present in the majority of the population [6]. Although the presence of disc degeneration was only weakly associated with LBP, the association with LBP increased with the severity of disc degeneration across disc levels [6]. Likewise, the severity of MRI finding at the SIJs defining a ‘SpA positive MRI’ is reported to influence the diagnostic value [7]. Focusing on the presence of individual MRI findings may therefore oversimplify the complexity of the degenerative and inflammatory axial processes and the interactions between various MRI findings, with the risk of overlooking potentially important clinical information.

In studies of MRI findings and LBP, there has been a focus on spinal degenerative MRI findings, while the SIJs have traditionally been the focus for studies of SpA. Recent research, however, indicates that MRI findings at the SIJs, previously thought to be indicative of SpA, are prevalent in patients with non-specific LBP [7, 8]. Also, spinal MRI findings associated with SpA can be difficult to distinguish from common degenerative vertebral endplate signal changes, which complicate the assessment of SpA [9]. Further to this, degenerative and SpA-related MRI research findings are most often reported in separate studies and in different study populations, which potentially reduces their applicability to daily clinical practice. There is therefore a need to explore the co-existence of degenerative and SpA-related MRI findings in both the spine and SIJs and their association with the clinical presentation of LBP. There may also be more than one pattern of co-existent MRI findings (subgroups), each of which has a different association with LBP.

Latent class analysis (LCA) is a multivariable statistical technique that attempts to find the subgroup structure which maximises the between-subgroup variance and minimises the within-subgroup variance, as a means to best explain the overall variance in the data. LCA has a number of advantages over traditional statistical clustering techniques, including greater classification accuracy, more precise metrics of subgroup model performance, the provision of posterior probabilities for subgroups and for individuals in each subgroup as a measure of subgroup model certainty, and the ability to manage variables of all data types (dichotomous, ordinal and continuous) [10, 11]. This novel statistical method therefore offers the possibility to explore patterns of MRI findings with the potential of identifying clinically important subgroups.

The objectives of this explorative study were: to investigate whether meaningful subgroups of patients with persistent LBP could be identified, based on MRI findings of the spine and SIJs, using LCA; and to investigate whether these subgroups differ in their demographic and clinical characteristics.

## Methods

### Study sample

**Fig. 1:**
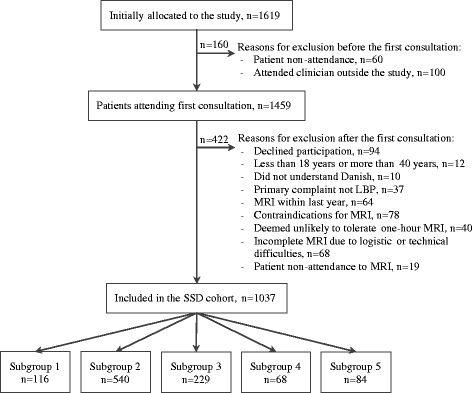
Flow of patients from entering the department to allocation into subgroups in the study. *LBP* low back pain, *MRI* magnetic resonance imaging, *SSD* Spines of Southern Denmark

Data for this study were from the ‘Spines of Southern Denmark’ cohort, which was established to investigate the use of MRI findings in the diagnoses of LBP and SpA. Detailed descriptions of this cohort have been published elsewhere [12]. Briefly, the cohort consists of 1037 patients with persistent LBP, with data from whole-spine and SIJ MRI scans, self-reported LBP questionnaires and analysis of blood samples. Patients were recruited from the Spine Centre of Southern Denmark, which is an outpatient, non-surgical unit specialising in the assessment of patients with back pain within a secondary care public hospital setting. During the study period, the criteria used to refer patients to the Spine Centre were an episode of back pain 2–12 months in duration and insufficient clinical response to conservative treatment in primary care. In a consecutive manner, secretaries responsible for the booking of appointments randomly allocated Caucasian patients to the project if they were aged 18–40 years and were referred with LBP, regardless of whether or not they had sciatica (see Fig. 1 for details).

### Magnetic resonance imaging

The MRI scanning protocol has been published previously [13]. In brief, an MRI scan of the whole spine and SIJs was performed with a 1.5 T MRI System (Philips Achieva, Best, the Netherlands) using a spine coil. For the spine, the following sequences were used: sagittal short-tau inversion recovery (STIR) and sagittal T1-weighted turbo spin-echo (TSE). An additional 3D volume isotropic T2-weighted acquisition sequence and an axial T2-weighted TSE sequence were performed for the lumbar spine. For the SIJ, the following three sequences were used: semi-coronal T1-weighted TSE, semi-coronal T1-weighted acquisition with spectral pre-saturation inversion recovery and semi-axial T2-weighted STIR. Three senior consultant radiologists, specialists in musculoskeletal imaging and SpA, participated in the research evaluation of the MRI scans. They were blinded to all clinical information, but not from the patients’ gender and age. Each MRI was evaluated by a single radiologist, except for cases where some uncertainty existed, which were resolved via consensus (6 % of the evaluations).

### MRI variables

For the evaluation of the spine, each intervertebral disc, vertebral endplate and subjacent bone marrow area, for 23 disc levels from C2–C3 to L5–S1, was assessed separately for the following MRI findings that were included in the current study: vertebral endplate signal changes of three types (bone marrow oedema, fatty marrow deposition and mixed type—an area with both bone marrow oedema and fatty marrow deposition), disc degeneration, disc protrusions, disc herniations and vertebral corner lesions.

Each SIJ was subdivided into four osseous locations: the cartilaginous and ligamentous portion of the iliac and the sacral bones (eight regions in total) [14]. The SIJs were assessed for the following periarticular findings: bone marrow oedema, fatty marrow deposition, erosions and sclerosis.

Details of the MRI evaluation have been published previously [13].

Because of the high number of MRI variables, to allay concerns about ‘overfitting’ the available data and to improve the clinical interpretability of the findings, data reduction was performed by the creation of sum scores. Seven spinal and four SIJ sum scores were generated from the assessed MRI findings. Sum scores for each of the three types of vertebral endplate signal changes and ‘corner lesions’ were generated by summing the number of relevant lesions in the 46 (2 × 23) assessed vertebral endplates (each with a possible range from 0 to 46); sum scores for ‘disc degeneration’ were generated by summing the original ordinal scale (from 0 to 3) that reported the severity of the lesion at the 23 assessed discs (possible range from 0 to 69); sum scores for ‘protrusions’ and ‘herniations’ were generated by summing the number of relevant lesions in the assessed discs (each with a possible range from 0 to 23); and sum scores for the four SIJ findings were generated by summing the original ordinal scale (from 0 to 3) that reported the size of the lesions at the eight assessed regions (possible range from 0 to 24 for each of the four SIJ sum scores).

### Reproducibility of the MRI variables

The MRI evaluation protocol has previously been tested for inter-observer and intra-observer agreement [13]. The MRI findings included in the current study had kappa values of more than 0.6, with the exception of vertebral corner lesion and erosions at the SIJ, which had kappa values for inter-observer agreement of 0.53 and 0.57, respectively [13].

### Clinical and biochemical data

Demographic and clinical characteristics were collected using patient self-reported questionnaires as part of the Spine Centre’s standard procedure. Details of this procedure have been reported previously [15]. The questionnaires were completed prior to the MRI scan. Blood samples were analysed for human leukocyte antigen (HLA)-B27 and high-sensitivity C-reactive protein (hsCRP).

The following clinical and demographic variables were included in the current study: age, sex, regular employment (being employed without public benefits and not a student or retired person), sick leave (persons in regular employment who reported sick leave due to LBP within the previous 3 months), being overweight (body mass index (BMI) > 25), smoking (one or more cigarettes daily), general health (EuroQol visual analogue scale [16]), back pain-related activity limitation (Roland Morris Disability Questionnaire [17] calculated as a proportional score, 0 % = no activity limitation/100 % = maximum activity limitation [18]), previous LBP episode (yes/no), LBP intensity (average of three 0–10 numerical rating scales of current LBP, worst LBP in the last 14 days and typical LBP in the last 14 days [19]), buttock pain (did you ever have buttock pain, yes /no), leg pain (defined as a person having indicated on a pain drawing his/her pain to include the anterior or posterior thigh, the calf and/or foot; and whose average leg pain intensity was ≥1 when measured the same way as for LBP intensity [19]), severe leg pain (leg pain intensity score > 3 [20]), pain in other areas (pain in areas other than that of the primary complaint over the last 2 weeks, yes/no), pregnancy-related LBP (onset of LBP related to recent pregnancy, yes /no), hsCRP level and HLA-B27 tissue typing (see Additional file 1 for reasoning behind the selection of these variables).

### Statistical analysis

The self-reported questionnaires and the coding from the MRI evaluations were entered directly into an electronic clinical registry (SpineData) via a browser-based evaluation form [15].

To identify subgroups of patients who had similar profiles of MRI findings, the data were analysed using LCA Latent Gold version 4.5 (Statistical Innovations, Belmont, MA, USA). The default settings of this software were used and the MRI sum scores were treated as ordinal data. LCA models with an increasing number of clusters were estimated until the best-fitting model was observed, identified as the model with the lowest Bayesian Information Criterion score. This model explains the most variance in the data while requiring the simplest specification of the model. However, to ensure an adequate size of the subgroups, no model was chosen with less than 5 % of the total sample size in a single subgroup. After identifying the best-fitting model, each individual’s posterior probability for each subgroup was calculated and the person was assigned to the subgroup with the highest posterior probability [21].

Differences in clinical characteristics between subgroups were tested using Kruskal–Wallis one-way analysis of variance. When subgroup differences were significant, pairwise comparisons were performed using chi-square tests for proportions and the Wilcoxon rank sum test for ordinal and continuous variables to identify the specific subgroups that differed. Significance level was set at 5 %. Subgroup differences were analysed using STATA 14.0 (StataCorp, College Station, TX, USA).

## Results

### LCA of MRI findings

**Fig. 2:**
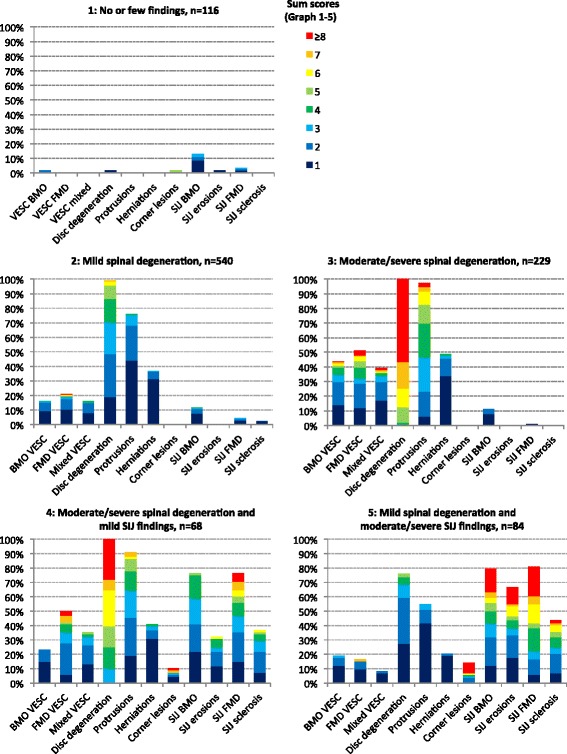
Prevalence of MRI findings in the five subgroups. *Bar height* represents the prevalence of patients with a given MRI finding. *Bar colour* reflects the sum scores for a given MRI finding as indicated. The sum scores reflect the cumulative ‘burden’ of a given MRI finding: sum scores for ‘VESC BMO’, ‘VESC FMD’, ‘VESC mixed’ and ‘corner lesions’ are the total number of lesions in all vertebral endplates; sum scores for ‘disc degeneration’ are the sum of an ordinal scale (0–3) reflecting the severity of lesions in all discs; and sum scores for ‘protrusions’ and ‘herniations’ are the total number of respective lesions in all discs. The four SIJ findings’ sum scores are the sum of ordinal scales (0–3) that reflect size of lesions at all SIJ regions. See Methods for details. *BMO* bone marrow oedema, *VESC* vertebral endplate signal changes, *FMD* fatty marrow deposition, *SIJ* sacroiliac joint (Colour figure online)

The LCA revealed a five-class model with: 116 patients (11 %) in Subgroup 1, 540 patients (54 %) in Subgroup 2, 229 patients (23 %) in Subgroup 3, 68 patients (7 %) in Subgroup 4 and 84 (8 %) patients in Subgroup 5. The prevalence of the MRI findings in each of the subgroups is presented in Fig. 2.

In general terms, the profile of each subgroup can be described in the following way. Patients in Subgroup 1 had no or few MRI findings and therefore were labelled ‘No or few findings’. Patients in Subgroup 2 had low sum scores on the variables related to spinal degeneration, with no or very few findings at the SIJs, and therefore were labelled ‘Mild spinal degeneration’*.* Patients in Subgroup 3 had higher sum scores on the variables related to spinal degeneration than Subgroup 2, with no or very few findings at the SIJs, and therefore were labelled ‘Moderate/severe spinal degeneration’. Patients in Subgroup 4 had similar sum scores on the variables related to spinal degeneration as Subgroup 3, but also MRI findings at the SIJ, and therefore were labelled ‘Moderate/severe spinal degeneration and mild SIJ findings’*.* Patients in Subgroup 5 had lower sum scores of the variables related to spinal degeneration than Subgroup 4, but higher sum scores of findings at the SIJs, and therefore were labelled ‘Mild spinal degeneration and moderate/severe SIJ findings’ (see Fig. 2 for details).

### Between-group differences in demographic and clinical characteristics

Significant differences between subgroups were found for four of the tested demographic variables (age, gender, being overweight and regular employment) and four of the clinical variables (previous LBP episodes, activity limitation, hsCRP and HLA-B27). The prevalence rates for these eight variables among the five MRI subgroups are presented in Table 1 (see Additional file 2 for the prevalence rates of all the tested variables).

**Table 1:** Prevalence rates of demographic and clinical characteristics in subgroups of MRI findings

	All	Subgroup 1: No or few findings	Subgroup 2: Mild spinal degeneration	Subgroup 3: Moderate/severe spinal degeneration	Subgroup 4: Moderate/severe spinal degeneration and mild SIJ findings	Subgroup 5: Mild spinal degeneration and moderate/severe SIJ findings	*P* value, significant pair-wise comparisons
	(*n* = 1037)	(*n* = 116)	(*n* = 540)	(*n* = 229)	(*n* = 68)	(*n* = 84)	
Age (years), median (IQR)	33 (27–37)	28 (23–33)	32 (26–36)	36 (32–39)	34 (30–37)	29 (25–33)	<0.05 for all comparisons except 1 vs 5 and 3 vs 4
Women,% (95 % CI)	54 (51–57)	76 (68–84)	51 (47–55)	40 (33–46)	68 (56–79)	68 (58–78)	< 0.01 for 2 vs all and 3 vs all
Regular employment, % (95 % CI)	70 (68–73)	67 (58–76)	71 (67–75)	76 (71–82)	56 (43–68)	67 (56–78)	< 0.05 for 2 vs 4 and 3 vs 4
Being overweight (BMI > 25), % (95 % CI)	53 (50–57)	36 (26–46)	51 (47–56)	63 (56–69)	67 (55–79)	53 (41–65)	< 0.05 for 1 vs all, 2 vs 3 and 2 vs 4
Previous LBP episode(s), % (95 % CI)	74 (72–77)	58 (48–67)	73 (69–76)	83 (79–88)	81 (71–91)	79 (70–89)	< 0.01 for 1 vs all and 2 vs 3
Activity limitation (RMDQ), median (IQR)	57 (39–74)	48 (35–74)	57 (39–74)	57 (39–74)	65 (48–78)	65 (48–78)	< 0.05 for 1 vs 4, 1 vs 5, 2 vs 4, 2 vs 5, 3 vs 4 and 3 vs 5
hsCRP, median (IQR)	1 (0–3)	1 (0–3)	1 (0–3)	1 (0–3)	2 (1–5)	3 (0–6)	< 0.05 for 1 vs 4, 1 vs 5, 2 vs 4, 2 vs 5, 3 vs 4 and 3 vs 5
HLA-B27-positive, % (95 % CI)	10 (9–12)	12 (6–18)	7 (5–10)	6 (3–9)	12 (4–20)	40 (29–50)	< 0.05 for 5 vs all, 1 vs 3 and 2 vs 4

Only variables with a statistically significant group difference (*P* < 0.05) were included in the above pair-wise comparison, *n* varies due to missing values

*MRI* magnetic resonance imaging, *IQR* interquartile range, *CI* confidence interval, *BMI* body mass index, *LBP* low back pain, *RMDQ* Roland Morris Disability Questionnaire (calculated as a proportional score (0 % = no activity limitation; 100 % = maximum activity limitation), *hsCRP* high-sensitivity C-reactive protein, *HLA* human leukocyte antigen

The two subgroups with MRI findings at the SIJs (Subgroups 4 and 5) had a higher activity limitation score and a higher level of hsCRP, compared with the three other subgroups. Previous LBP episodes and being overweight were also more common among patients with MRI findings at the SIJs compared with patients with no or few MRI findings (Subgroup 1). Furthermore, the two SIJ subgroups had a higher prevalence of women compared with the two subgroups with spinal degeneration (Subgroups 2 and 3). The two SIJ subgroups differed according to age and prevalence of HLA-B27-positive status: Subgroup 5 was younger and had a higher prevalence of HLA-B27-positive status compared with patients in Subgroup 4.

Across Subgroups 1–3, median age, prevalence rates of women, being overweight and reports of previous LBP episodes were lowest in Subgroup 1, higher in Subgroup 2 and highest in Subgroup 3 (see Table 1 for details).

### Post-hoc analysis

A post-hoc analysis was performed to analyse a potential association between gender and HLA-B27 in each of the subgroups. In Subgroup 5, men were more likely to be HLA-B27-positive than women: prevalence rate in men 59 % (95 % CI: 39–79) versus 30 % (17–42) in women (*P* < 0.05). No statistically significant differences in the prevalence of HLA-B27 were found between genders either in any of the other subgroups or in the total sample.

Furthermore, to investigate possible confounding by age and gender of the association between the MRI subgroups and being overweight and previous LBP episodes, respectively, we performed post-hoc analyses using logistic regression analyses. The statistically significant associations identified in the univariate analyses between the MRI subgroups and previous LBP episodes and being overweight remained statistically significant in the post-hoc analyses when adjusted for age and gender, with the exception of the difference in the prevalence of being overweight in Subgroup 2 and Subgroup 3 (*P* = 0.07). Possible confounding by gender and being overweight regarding the associations between the MRI subgroups and hsCRP levels were similarly analysed using logistic regression. After adjustment for gender and BMI, there was no statistically significant difference in hsCRP levels between Subgroup 4 and Subgroups 1–3 (*P* > 0.3). When adjusted, the difference remained statistically significant for Subgroup 5 compared with Subgroups 2 and 3 (*P* < 0.01), but not when comparing Subgroups 5 and 1 (*P* = 0.08) (data not shown).

## Discussion

Using LCA, we identified five subgroups with differing severity of spinal and SIJ MRI findings, and several interesting associations between these MRI subgroups and demographic and clinical characteristics were found. The following discussion considers why these clinical aspects of MRI findings in the spine and SIJs might be meaningful clinically.

Firstly, noteworthy differences were observed between the two SIJ subgroups: one subgroup had moderate SIJ findings in combination with moderate to severe spinal degeneration (Subgroup 4), and the other had severe findings at the SIJs but only mild spinal degeneration (Subgroup 5). There might be two different causes of these SIJ MRI findings: one cause being age-related and load-related degeneration, which is likely to be most prevalent in Subgroup 4; and the other cause being SpA, which is likely to be most prevalent in Subgroup 5. This hypothesis was supported by a younger mean age in combination with a higher prevalence of HLA-B27-positive status in Subgroup 5 compared with Subgroup 4. These results thus suggest that, in addition to SpA, SIJ findings, including bone marrow oedema, can be part of degenerative age-related processes. This is further supported by the observation that the association between bone marrow oedema and age-related and load-related degeneration is well established in other body regions; that is, the spine, hip and knee joints [22–24].

Moreover, the results from the current study suggest other possible causes of MRI findings at the SIJs, beyond SpA. Firstly, we found noteworthy differences in the gender distribution across the identified MRI subgroups. While the patients in the two SIJ subgroups were more often women, the male gender was quite strongly associated with spinal degenerative findings. This could be explained by a different load distribution on the axial skeleton in men and women. Also, pregnancy and birth have been suggested to cause pathoanatomical changes at the SIJs that can be observed using MRI [25]. We have previously found associations between pregnancy-related back pain and the presence of SIJ bone marrow oedema, sclerosis and erosions in the current study sample [26]. Hence, it is possible that the overrepresentation of women in the SIJ subgroups could be explained by an association between some of the SIJ MRI findings and pregnancy, although in the current study the subgroups with SIJ findings were not found to be associated with reports of pregnancy-related back pain. Furthermore, men with moderate to severe SIJ MRI findings were more likely to be HLA-B27-positive compared with women with moderate to severe SIJ MRI findings (59 % versus 30 %). Thus, it seems possible that men with moderate to severe SIJ MRI findings have a higher risk of having SpA compared with women with these MRI findings. The association between men and spinal degenerative findings has been reported previously [27, 28] and could be explained by heavy work being more frequent among Danish men compared with women [29]. However, it is also possible that men simply have a higher risk of spinal degeneration.

Secondly, being overweight seems to influence the MRI findings at the SIJs as well as the spinal MRI findings, because the prevalence of being overweight was significantly lower in patients with no or few MRI findings compared with the subgroups with spinal degeneration and/or SIJ findings, regardless of age and gender. This result may be due to the extra load on the axial skeleton caused by being overweight, resulting in an acceleration of degenerative changes in both the spine and the SIJ. The association between being overweight and vertebral endplate signal changes and disc degeneration has been reported previously [28, 30, 31], while the association between being overweight and SIJ findings, we believe, has not been investigated previously. The association between being overweight and osteoarthritis in general is well established [32]. However, further studies are needed to investigate the link between being overweight and the presence of pain-generating pathoanatomical changes in the spine and SIJ, because this might constitute an increasing public health problem due to increasing obesity rates [33].

Traditionally, the presence of subchondral or periarticular bone marrow oedema at the SIJs has been considered specific for SpA [34] and is one of the cornerstones in the Assessment in SpondyloArthritis International Society (ASAS) criteria for SpA [35]. However, recent studies have shown that subchondral or periarticular SIJ bone marrow oedema lesions are also prevalent in non-SpA patients, which has led to debate about the diagnostic value of this MRI finding [36]. The results from the current cohort study adds to this debate by suggesting that SIJ findings, including bone marrow oedema, can be part of degenerative age-related processes and are also associated with female gender and being overweight; they are not only seen as a part of the SpA disease entity.

Another interesting finding from our study was that both of the two SIJ subgroups (Subgroups 4 and 5) had more back pain-related activity limitation compared with the other subgroups. These results indicate that patients with SIJ MRI findings are more disabled, with the between-group difference approximating the threshold that patients rate in this secondary care setting as important (minimal clinical importance difference of 9 on a 0–100 scale) [37]. SIJ involvement may cause greater disability compared with spinal involvement due to the biomechanical involvement of the SIJs in gait and weight-bearing functions. Because the prevalence of being overweight also varied across the MRI subgroups and was highest in people with the most severe MRI findings, both spinal and SIJ, being overweight could be causally related to the presence of severe axial MRI findings and subsequent back pain-related activity limitation. However, because cross-sectional data are inappropriate for exploring causal and temporal relationships, further investigation of this relationship would require longitudinal data.

Also interesting was that the prevalence of self-reported previous LBP episodes was notably lower among patients with no or few MRI findings compared with all other subgroups, irrespective of age and gender. This result suggests that patients with spinal degeneration and/or SIJ findings may have a more recurrent pain course compared with patients without MRI findings. This theory is supported by an earlier study reporting a positive association between the presence of disc degeneration and previous LBP episodes [38]. However, further longitudinal investigations are needed to elaborate the importance of the severity of MRI findings to the disease course.

The methodological strengths of this study are, firstly, the inclusion of MRI analysis of both the SIJs and the whole spine, making data-driven exploration of subgroups of MRI findings in the most important regions of the axial skeleton possible in one study. Furthermore, the standardised MRI protocol used in this study increases the integrity and uniformity of the data. In addition, all of the clinical and demographic variables tested in the analysis were chosen a priori and the rationale for including each of them was described. Finally, the large number of participants strengthens the prevalence estimates obtained.

There are also important limitations to the current study that should be taken into consideration when interpreting the results. Firstly, because the high number of variables tested increases the risk of chance findings due to mass significance, between-subgroup comparisons should be interpreted as hypothesis-generating only. Because of the explorative nature of the study and, to minimise the risk of overlooking potential important associations (type 2 errors) no conservative statistical corrections were made. Thus, the results should be confirmed in subsequent studies before definitive conclusions can be drawn. Furthermore, the MRI evaluations were not blinded for age and gender. Moreover, the age cut-off value was set at 40 years and consequently the age interval was quite narrow, which might obscure possible associations with age. Also, it is possible that other subgroups would be identified in different cohorts. Furthermore, the subgroups resulting from LCA are determined by the variables chosen for the analysis. Therefore, while we attempted to choose the MRI variables used in the LCA based on our own conceptual model as well as previous studies, the inclusion of other MRI variables, such as nerve root involvement, should be considered in future studies and might modify the subgroups found. Lastly, further investigations are needed to assess any prognostic and treatment response implications of the identified MRI subgroups.

## Conclusions

In summary, five clinically interpretable subgroups were identified based on the LCA of MRI findings of the spine and SIJs. The demographic and clinical differences between the identified subgroups contribute in the following ways to an aetiological understanding of these MRI findings and their role in the clinical presentation of back pain. Firstly, the results indicate that findings at the SIJs not only can be seen as part of the SpA disease entity, but are also associated with gender, being overweight and age-related degeneration. Moreover, the results indicate that patients with SIJ findings, regardless of cause, are more disabled compared with patients without MRI findings at the SIJs. Finally, the results indicate that patients with spinal degeneration and/or SIJ findings may have a more recurrent pain course compared with LBP patients without MRI findings.

## Additional files

Additional file 1: Reasoning underpinning the selection of clinical and demographic characteristics. (PDF 97 kb)
Additional file 2: Prevalence rates of all tested demographic and clinical characteristics in the subgroups of MRI findings. (PDF 92 kb)

## Abbreviations

BMI: Body mass index
HLA: Human leukocyte antigen
hsCRP: High-sensitivity C-reactive protein
LBP: Low back pain
LCA: Latent class analysis
MRI: Magnetic resonance imaging
SIJ: Sacroiliac joint
SpA: Spondyloarthritis
STIR: Short-tau inversion recovery
TSE: Turbo spin-echo

## References

[1] Vos T, Flaxman AD, Naghavi M, Lozano R, Michaud C, Ezzati M, Shibuya K, Salomon JA, Abdalla S, Aboyans V (2012). Years lived with disability (YLDs) for 1160 sequelae of 289 diseases and injuries 1990-2010: a systematic analysis for the Global Burden of Disease Study 2010. Lancet.

[2] Endean A, Palmer KT, Coggon D (2011). Potential of magnetic resonance imaging findings to refine case definition for mechanical low back pain in epidemiological studies: a systematic review. Spine (Phila Pa 1976).

[3] Chou D, Samartzis D, Bellabarba C, Patel A, Luk KD, Kisser JM, Skelly AC (2011). Degenerative magnetic resonance imaging changes in patients with chronic low back pain: a systematic review. Spine (Phila Pa 1976).

[4] Jensen TS, Karppinen J, Sorensen JS, Niinimaki J, Leboeuf-Yde C (2008). Vertebral endplate signal changes (Modic change): a systematic literature review of prevalence and association with non-specific low back pain. Eur Spine J.

[5] Arnbak B, Leboeuf-Yde C, Jensen TS (2012). A systematic critical review on MRI in spondyloarthritis. Arthritis Res Ther.

[6] Cheung KM, Karppinen J, Chan D, Ho DW, Song YQ, Sham P, Cheah KS, Leong JC, Luk KD (2009). Prevalence and pattern of lumbar magnetic resonance imaging changes in a population study of one thousand forty-three individuals. Spine (Phila Pa 1976).

[7] Weber U, Ostergaard M, Lambert RG, Pedersen SJ, Chan SM, Zubler V, Rufibach K, Zhao Z, Maksymowych WP (2015). Candidate lesion-based criteria for defining a positive sacroiliac joint MRI in two cohorts of patients with axial spondyloarthritis. Ann Rheum Dis.

[8] Weber U, Lambert RG, Ostergaard M, Hodler J, Pedersen SJ, Maksymowych WP (2010). The diagnostic utility of magnetic resonance imaging in spondylarthritis: an international multicenter evaluation of one hundred eighty-seven subjects. Arthritis Rheum.

[9] Hermann KG, Baraliakos X, van der Heijde DM, Jurik AG, Landewe R, Marzo-Ortega H, Ostergaard M, Rudwaleit M, Sieper J, Braun J (2012). Descriptions of spinal MRI lesions and definition of a positive MRI of the spine in axial spondyloarthritis: a consensual approach by the ASAS/OMERACT MRI study group. Ann Rheum Dis.

[10] Magidson J, Vermunt J (2002). Latent class models for clustering: a comparison with K-means. Can J Market Res.

[11] Wallace CS (2005). Statistical and Inductive Inference by Minimum Message Length.

[12] Arnbak B, Grethe Jurik A, Horslev-Petersen K, Hendricks O, Hermansen LT, Loft AG, Ostergaard M, Pedersen SJ, Zejden A, Egund N (2016). Associations between spondyloarthritis features and magnetic resonance imaging findings: a cross-sectional analysis of 1,020 patients with persistent low back pain. Arthritis Rheumatol.

[13] Arnbak B, Jensen TS, Manniche C, Zejden A, Egund N, Jurik AG (2013). Spondyloarthritis-related and degenerative MRI changes in the axial skeleton—an inter- and intra-observer agreement study. BMC Musculoskelet Disord.

[14] Madsen KB, Jurik AG (2010). Magnetic resonance imaging grading system for active and chronic spondylarthritis changes in the sacroiliac joint. Arthritis Care Res (Hoboken).

[15] Kent P, Kongsted A, Jensen TS, Albert HB, Schiottz-Christensen B, Manniche C (2015). SpineData—a Danish clinical registry of people with chronic back pain. Clin Epidemiol.

[16] EuroQol G (1990). EuroQol—a new facility for the measurement of health-related quality of life. Health Policy.

[17] Roland M, Morris R (1983). A study of the natural history of back pain. Part I: development of a reliable and sensitive measure of disability in low-back pain. Spine (Phila Pa 1976).

[18] Kent P, Lauridsen HH (2011). Managing missing scores on the Roland Morris Disability Questionnaire. Spine (Phila Pa 1976).

[19] Manniche C, Asmussen K, Lauritsen B, Vinterberg H, Kreiner S, Jordan A (1994). Low Back Pain Rating scale: validation of a tool for assessment of low back pain. Pain.

[20] Gerbershagen HJ, Rothaug J, Kalkman CJ, Meissner W (2011). Determination of moderate-to-severe postoperative pain on the numeric rating scale: a cut-off point analysis applying four different methods. Br J Anaesth.

[21] Nylund KL, Asparouhov T, Muthén BO (2007). Deciding on the number of classes in latent class analysis and growth mixture modeling: a Monte Carlo simulation study. Struct Equation Model.

[22] Jensen TS, Bendix T, Sorensen JS, Manniche C, Korsholm L, Kjaer P (2009). Characteristics and natural course of vertebral endplate signal (Modic) changes in the Danish general population. BMC Musculoskelet Disord.

[23] Taljanovic MS, Graham AR, Benjamin JB, Gmitro AF, Krupinski EA, Schwartz SA, Hunter TB, Resnick DL (2008). Bone marrow edema pattern in advanced hip osteoarthritis: quantitative assessment with magnetic resonance imaging and correlation with clinical examination, radiographic findings, and histopathology. Skeletal Radiol.

[24] Lim YZ, Wang Y, Wluka AE, Davies-Tuck ML, Teichtahl A, Urquhart DM, Cicuttini FM (2013). Are biomechanical factors, meniscal pathology, and physical activity risk factors for bone marrow lesions at the knee? A systematic review. Semin Arthritis Rheum.

[25] Eshed I, Miloh-Raz H, Dulitzki M, Lidar Z, Aharoni D, Liberman B, Lidar M (2015). Peripartum changes of the sacroiliac joints on MRI: increasing mechanical load correlating with signs of edema and inflammation kindling spondyloarthropathy in the genetically prone. Clin Rheumatol.

[26] Arnbak B, Jensen TS, Egund N, Zejden A, Horslev-Petersen K, Manniche C, Jurik AG (2016). Prevalence of degenerative and spondyloarthritis-related magnetic resonance imaging findings in the spine and sacroiliac joints in patients with persistent low back pain. Eur Radiol.

[27] Kjaer P, Leboeuf-Yde C, Korsholm L, Sorensen JS, Bendix T (2005). Magnetic resonance imaging and low back pain in adults: a diagnostic imaging study of 40-year-old men and women. Spine (Phila Pa 1976).

[28] Arana E, Kovacs FM, Royuela A, Estremera A, Asenjo B, Sarasibar H, Amengual G, Galarraga I, Alonso A, Casillas C (2011). Modic changes and associated features in Southern European chronic low back pain patients. Spine J.

[29] Christensen AI, Ekholm O, Davidsen M, Juel K (2012). Sundhed og sygelighed i Danmark 2010 & udviklingen siden 1987.

[30] Mok FP, Samartzis D, Karppinen J, Fong DY, Luk KD, Cheung KM (2016). Modic changes of the lumbar spine: prevalence, risk factors, and association with disc degeneration and low back pain in a large-scale population-based cohort. Spine J.

[31] Liuke M, Solovieva S, Lamminen A, Luoma K, Leino-Arjas P, Luukkonen R, Riihimaki H (2005). Disc degeneration of the lumbar spine in relation to overweight. Int J Obes (Lond).

[32] Lidstone JS, Ells LJ, Finn P, Whittaker VJ, Wilkinson JR, Summerbell CD (2006). Independent associations between weight status and disability in adults: results from the Health Survey for England. Public Health.

[33] Di Cesare M, Bentham J, Stevens GA, Zhou B, Danaei G, Lu Y, Bixby H, Cowan MJ, Riley LM, Collaboration NCDRF (2016). Trends in adult body-mass index in 200 countries from 1975 to 2014: a pooled analysis of 1698 population-based measurement studies with 19.2 million participants. Lancet.

[34] Rudwaleit M, Jurik AG, Hermann KG, Landewe R, van der Heijde D, Baraliakos X, Marzo-Ortega H, Ostergaard M, Braun J, Sieper J (2009). Defining active sacroiliitis on magnetic resonance imaging (MRI) for classification of axial spondyloarthritis: a consensual approach by the ASAS/OMERACT MRI group. Ann Rheum Dis.

[35] Rudwaleit M, van der Heijde D, Landewe R, Listing J, Akkoc N, Brandt J, Braun J, Chou CT, Collantes-Estevez E, Dougados M (2009). The development of Assessment of SpondyloArthritis International Society classification criteria for axial spondyloarthritis (part II): validation and final selection. Ann Rheum Dis.

[36] Weber U, Jurik AG, Lambert RG, Maksymowych WP (2016). Imaging in spondyloarthritis: controversies in recognition of early disease. Curr Rheumatol Rep.

[37] Lauridsen HH, Hartvigsen J, Manniche C, Korsholm L, Grunnet-Nilsson N (2006). Responsiveness and minimal clinically important difference for pain and disability instruments in low back pain patients. BMC Musculoskelet Disord.

[38] Videman T, Battie MC, Gibbons LE, Maravilla K, Manninen H, Kaprio J (2003). Associations between back pain history and lumbar MRI findings. Spine (Phila Pa 1976).

