# Supplementary material for: Identification of subgroups of inflammatory and degenerative MRI findings in the spine and sacroiliac joints: a latent class analysis of 1037 patients with persistent low back pain
Source: Arthritis Res Ther. 2016 Oct 13;18:237. doi: 10.1186/s13075-016-1131-x (PMC5062874; doi:10.1186/s13075-016-1131-x)
Supplement: Additional file 1: — Reasoning underpinning the selection of clinical and demographic characteristics. (PDF 97 kb) [file 13075_2016_1131_MOESM1_ESM.pdf]

## Additional file 1. Reasoning underpinning the selection of clinical and demographic characteristics

| Clinical and demographic characteristics | Reasons for inclusion |
|---|---|
| **Age** | Degenerative changes are known to be age-related [1, 2] |
| **Sex** | Ankylosing spondylitis is associated with male gender [3]. Furthermore, the anatomy and load-distribution through the axial skeleton vary between genders [4], with a possible influence on the prevalence of load-related MRI findings. |
| **Regular employment** (being employed without public benefits and not a student or retired) | Severe spinal degeneration or spondyloarthritis may be associated with reduced work capacity [5, 6]. |
| **Sick leave** due to back pain in the last 3 months for patients with regular employment | Severe spinal degeneration or spondyloarthritis may be associated with reduced work capacity [5, 6]. |
| **Being overweight** (BMI ≥25) | Being overweight has been associated with vertebral endplate signal changes [1] and disc degeneration [7]. |
| **Smoking** (one or more cigarettes daily) | Smoking is known to delay healing and thus smoking might influence the presence of MRI findings linked to tissue damage such as bone marrow oedema and/or disc herniations. Smoking has also been shown to be a risk factor for vertebral endplate signal changes [1, 8]. |
| **General health**, (EuroQol visual analogue scale [9]) | Hypothesis: Severe spinal degeneration or spondyloarthritis may be associated with low general health. |
| **Activity limitation** (RMDQ [10] calculated as a proportional score (0% = no activity limitation; 100% = maximum activity limitation [11]) | Hypothesis: Severe spinal degeneration or spondyloarthritis may be associated with activity limitation. LBP patients with vertebral endplate signal changes have previously been reported to have more activity limitation at one year follow-up than LBP patients without vertebral endplate signal changes [6]. |
| **Previous LBP episode(s)** | A previous study has reported an association between disc degeneration and previous episodes of LBP [12]. Furthermore, spondyloarthritis is most often a chronic disease, and patients with MRI findings related to spondyloarthritis might be more likely to have previous LBP episodes. |
| **LBP intensity** (averaged on 0–10 Numerical Rating Scales of present LBP, worst LBP last 14 days and typical LBP last 14 days [13]) | Hypothesis: Severe spinal degeneration or spondyloarthritis may be associated with higher LBP intensity. |
| **Buttock pain** (yes/no to the question : 'Have you had buttock pain) | Hypothesis: Patients with severe MRI findings at the SIJ may be more likely to have buttock pain, as SIJ are known to produce referred leg pain [14]. |
| **Leg pain** (indicated on pain drawing) | Patients with disc herniations might have leg pain as a result of nerve root compression or somatic referred leg pain. Furthermore, the SIJ are known to produce referred leg pain [14]. |
| **Severe leg pain** (Intensity of leg pain measured the same way as for LBP intensity, with a score >3 ) | Hypothesis: The intensity of leg pain may vary with the cause of leg pain (referred pain from the SIJ, nerve root compression due to herniation, or somatic referred leg pain). |

Table continued

| Clinical and demographic characteristics | Reasons for inclusion |
|---|---|
| **Pain in other areas** (yes/ no to the question: Over the last 2 weeks, have you been bothered by pain in body parts other than you have marked on the previous drawing?) | Hypothesis: Degeneration/osteoarthritis might be a widespread condition affecting various anatomical regions. |
| **Pregnancy related LBP** (yes/ no to the question: Was the onset of your low back pain or leg pain (sciatica) associated with a recent or current pregnancy?) | Pregnancy-related changes of the pelvic girdle have been suggest to cause changes that can be visualised with MRI [15]. |
| **High sensitive C-reactive protein** | C-reactive protein has been associated with vertebral endplate signal changes [16]. Furthermore, elevated CRP levels are included in the diagnosis of spondyloarthritis [17]. |
| **HLA-B27** | HLA-B27 are central in the diagnosis of spondyloarthritis [17]. |

SIJ: Sacroiliac joint, BMI: Body mass index, MRI: Magnetic resonance imaging, VAS: Visual analogue scale, RMDQ: Roland Morris Disability Questionnaire (calculated as a proportional score (0% = no activity limitation; 100% = maximum activity limitation), LBP: Low back pain, HLA: Human leukocyte antigen

**Reference list**


1. Mok FP, Samartzis D, Karppinen J, Fong DY, Luk KD, Cheung KM: Modic changes of the lumbar spine: prevalence, risk factors, and association with disc degeneration and low back pain in a large-scale population-based cohort. Spine J. 2016;16:32-41.

2. Cheung KM, Karppinen J, Chan D, Ho DW, Song YQ, Sham P, Cheah KS, Leong JC, Luk KD: Prevalence and pattern of lumbar magnetic resonance imaging changes in a population study of one thousand forty-three individuals. Spine (Phila Pa 1976). 2009;34:934-40.

3. Gran JT, Husby G, Hordvik M: Prevalence of ankylosing spondylitis in males and females in a young middle-aged population of Tromso, northern Norway. Ann Rheum Dis. 1985;44:359-67.

4. Dietrichs E: Anatomy of the pelvic joints--a review. Scand J Rheumatol Suppl. 1991;88:4-6.

5. van der Weijden MA, Boonen A, van der Horst-Bruinsma IE: Problems in work participation and resource use should not be underestimated in patients with early spondyloarthritis. J Rheumatol. 2014;41:2413-20.

6. Jensen OK, Nielsen CV, Sorensen JS, Stengaard-Pedersen K: Type 1 Modic changes was a significant risk factor for 1-year outcome in sick-listed low back pain patients: a nested cohort study using magnetic resonance imaging of the lumbar spine. Spine J. 2014;14:2568-81.

7. Liuke M, Solovieva S, Lamminen A, Luoma K, Leino-Arjas P, Luukkonen R, Riihimaki H: Disc degeneration of the lumbar spine in relation to overweight. Int J Obes (Lond). 2005;29:903-8.

8. Patel RA, Wilson RF, Patel PA, Palmer RM: The effect of smoking on bone healing: A systematic review. Bone Joint Res. 2013;2:102-11.

9. EuroQol G: EuroQol - a new facility for the measurement of health-related quality of life. Health Policy. 1990;16:199-208.

10. Roland M, Morris R: A study of the natural history of back pain. Part I: development of a reliable and sensitive measure of disability in low-back pain. Spine (Phila Pa 1976). 1983;8:141-4.

11. Kent P, Lauridsen HH: Managing missing scores on the Roland Morris Disability Questionnaire. Spine (Phila Pa 1976). 2011;36:1878-84.

12. Videman T, Battie MC, Gibbons LE, Maravilla K, Manninen H, Kaprio J: Associations between back pain history and lumbar MRI findings. Spine (Phila Pa 1976). 2003;28:582-8.

13. Manniche C, Asmussen K, Lauritsen B, Vinterberg H, Kreiner S, Jordan A: Low Back Pain Rating scale: validation of a tool for assessment of low back pain. Pain. 1994;57:317-26.

14. Szadek KM, van der Wurff P, van Tulder MW, Zuurmond WW, Perez RS: Diagnostic validity of criteria for sacroiliac joint pain: a systematic review. J Pain. 2009;10:354-68.

15. Eshed I, Miloh-Raz H, Dulitzki M, Lidar Z, Aharoni D, Liberman B, Lidar M: Peripartum changes of the sacroiliac joints on MRI: increasing mechanical load correlating with signs of edema and inflammation kindling spondyloarthropathy in the genetically prone. Clin Rheumatol. 2015;34:1419-26.

16. Rannou F, Ouanes W, Boutron I, Lovisi B, Fayad F, Mace Y, Borderie D, Guerini H, Poiraudeau S, Revel M: High-sensitivity C-reactive protein in chronic low back pain with vertebral end-plate Modic signal changes. Arthritis Rheum. 2007;57:1311-5.

17. Rudwaleit M, van der Heijde D, Landewe R, Listing J, Akkoc N, Brandt J, Braun J, Chou CT, Collantes-Estevez E, Dougados M *et al*: The development of Assessment of SpondyloArthritis international Society classification criteria for axial spondyloarthritis (part II): validation and final selection. Ann Rheum Dis. 2009;68:777-83.
