# Supplementary material for: Identification of subgroups of inflammatory and degenerative MRI findings in the spine and sacroiliac joints: a latent class analysis of 1037 patients with persistent low back pain
Source: Arthritis Res Ther. 2016 Oct 13;18:237. doi: 10.1186/s13075-016-1131-x (PMC5062874; doi:10.1186/s13075-016-1131-x)
Supplement: Additional file 2: — Prevalence rates of all tested demographic and clinical characteristics in the subgroups of MRI findings. (PDF 92 kb) [file 13075_2016_1131_MOESM2_ESM.pdf]

Additional file 2. Prevalence rates of all tested demographic and clinical characteristics in the subgroups of MRI findings

	All	*1: No or few findings*	*2: Mild spinal degeneration*	*3: Moderate/severe spinal degeneration*	*4: Moderate/severe spinal degeneration and mild SIJ findings*	*5: Mild spinal degeneration and moderate/severe SIJ findings*	P-value
	n = 1037	*n = 116*	*n = 540*	*n = 229*	*n = 68*	*n =84*	Any group difference
**Age in years**, median (IQR)	33 (27-37)	28 (23-33)	32 (26-36)	36 (32-39)	34 (30-37)	29 (25-33)	0.0001*
**Women**, % (95%CI)	54 (51-57)	76 (68-84)	51 (47-55)	40 (33-46)	68 (56-79)	68 (58-78)	0.0001*
**Regular employment**, % (95%CI)	70 (68-73)	67 (58-76)	71 (67-75)	76 (71-82)	56 (43-68)	67 (56-78)	0.0197*
**Sick leave**, % (95%CI)	48 (44-51)	44 (32-56)	47 (41-52)	50 (42-0,58)	51 (34-69)	48 (33-63)	0.9031
**Being overweight**, (BMI>25) % (95%CI)	53 (50-57)	36 (26-46)	51 (47-56)	63 (56-69)	67 (55-79)	53 (41-65)	0.0001*
**Smoking**, % (95%CI)	30 (27-33)	37 (27-46)	29 (25-33)	27 (21-33)	36 (23-49)	30 (19-41)	0.3560
**General health (EuroQol VAS)**, median (IQR)	52 (37-74)	51 (39-71)	55 (39-75)	53 (36-74)	50 (34-68)	50 (34-63)	0.0758
**Activity limitation (RMDQ),** median (IQR)	57 (39-74)	48 (35-74)	57 (39-74)	57 (39-74)	65 (48-78)	65 (48-78)	0.0260*
**Previous LBP episode(s)**, % (95%CI)	74 (72-77)	58 (48-67)	73 (69-76)	83 (79-88)	81 (71-91)	79 (70-89)	0.0001*
**LBP intensity**[1], median (IQR)	6 (5-7)	6 (5-7)	6 (5-7)	6 (4-7)	6 (5-8)	7 (5-7)	0.2582
**Buttock pain**, % (95%CI)	82 (80-85)	74 (66-83)	84 (80-87)	81 (76-86)	87 (78-96)	84 (76-93)	0.1891
**Leg pain**, % (95% CI)	27 (24-30)	24 (15-32)	28 (24-33)	26 (20-32)	31 (18-44)	23 (13-34)	0.7395
**Severe leg pain**[2], % (95% CI)	42 (38-46)	31 (21-41)	42 (37-47)	41 (33-49)	54 (40-68)	51 (38-64)	0.0541
**Pain in other areas**, % (95%CI)	41 (38-44)	35 (25-44)	41 (37-45)	40 (33-46)	46 (33-59)	48 (36-60)	0.3997
**Pregnancy related LBP**, % (95%CI)	15 (12-18)	12 (5-20)	14 (10-19)	15 (7-22)	16 (5-27)	27 (14-39)	0.2393
**hsCRP,** median (IQR)	1 (0-3)	1 (0-3)	1 (0-3)	1 (0-3)	2 (1-5)	3 (0-6)	0.0004*
**HLA-B27 positive**, % (95% CI)	10 (9-12)	12 (6-18)	7 (5-10)	6 (3-9)	12 (4-20)	40 (29-50)	0.0001*

IQR: Inter-quartile range, BMI: Body mass index, VAS: Visual analogue scale, LBP: Low back pain, RMDQ: Roland Morris Disability Questionnaire (calculated as a proportional score (0% = no activity limitation; 100% = maximum activity limitation), hsCRP: high sensitive C-reactive protein, HLA: human leukocyte antigen
1: Averaged of 0–10 numerical rating scales on present LBP, worst LBP last 14 days and typical LBP last 14 days, 2: Intensity of leg pain measured the same way as for LBP intensity > 3, *Significance level was set at 5%, n varies due to missing values
